# Age-Related Reference Intervals of the Main Biochemical and Hematological Parameters in C57BL/6J, 129SV/EV and C3H/HeJ Mouse Strains

**DOI:** 10.1371/journal.pone.0003772

**Published:** 2008-11-20

**Authors:** Cristina Mazzaccara, Giuseppe Labruna, Gennaro Cito, Marzia Scarfò, Mario De Felice, Lucio Pastore, Lucia Sacchetti

**Affiliations:** 1 Dipartimento di Biochimica e Biotecnologie Mediche, Università degli Studi di Napoli Federico II and CEINGE-Biotecnologie Avanzate S.C.aR.L., Naples, Italy; 2 Biogem S.C.aR.L., Naples, Italy; Ordway Research Institute, United States of America

## Abstract

**Background:**

Although the mouse is the animal model most widely used to study the pathogenesis and treatment of human diseases, reference values for biochemical parameters are scanty or lacking for the most frequently used strains. We therefore evaluated these parameters in the C57BL/6J, 129SV/EV and C3H/HeJ mice.

**Methodology/Principal Findings:**

We measured by dry chemistry 26 analytes relative to electrolyte balance, lipoprotein metabolism, and muscle/heart, liver, kidney and pancreas functions, and by automated blood counter 5 hematological parameters in 30 animals (15 male and 15 female) of each mouse strain at three age ranges: 1–2 months, 3–8 months and 9–12 months. Whole blood was collected from the retro-orbital sinus. We used quality control procedures to investigate analytical imprecision and inaccuracy. Reference values were calculated by non parametric methods (median and 2.5^th^ and 97.5^th^ percentiles). The Mann-Whitney and Kruskal-Wallis tests were used for between-group comparisons. Median levels of GLU, LDH, Chol and BUN were higher, and LPS, AST, ALP and CHE were lower in males than in females (*p* range: 0.05–0.001). Inter-strain differences were observed for: (1) GLU, t-Bil, K^+^, Ca^++^, PO_4_^−^ (*p*<0.05) and for TAG, Chol, AST, Fe^++^ (*p*<0.001) in 4–8 month-old animals; (2) for CK, Crea, Mg^++^, Na^++^, K^+^, Cl^−^ (*p*<0.05) and BUN (*p*<0.001) in 2- and in 10–12 month-old mice; and (3) for WBC, RBC, HGB, HCT and PLT (*p*<0.05) during the 1 year life span.

**Conclusion/Significance:**

Our results indicate that metabolic variations in C57BL/6J, 129SV/EV and C3H/HeJ mice after therapeutic intervention should be evaluated against gender- and age-dependent reference intervals.

## Introduction

Mouse (*Mus Musculus*) is the animal model most widely used to study the pathogenesis and treatment of human diseases [1]. Mouse models have various advantages. First, the mouse is evolutionarily very close to humans (the split occurred 70 million years ago) and its embryonic development recapitulates many aspects of human development in terms of body structure formation and gene activation. The mouse is one of the smallest mammals and has a short generation time; this reduces the cost of animal experimentation and a large number of animals are available for study [2]. In addition, because embryonic stem cells were derived from mice over two decades ago, germ-line modification and knock-out mice have been generated for specific human diseases and developmental biology studies [1].

These models are usually generated in mixed strains (C57BL/6J and 129SV/EV) and then backcrossed into the required strains. Pure mice strains have been generated using brother/sister mating since the beginning of the twentieth century and have distinctive features as regards, reproductivity, behavioral characteristics and predisposition to specific diseases. Different mouse strains are used for different purposes. For instance, mice with reduced immune competence, such as C57BL/6J, are generally used in gene therapy experiments to obtain proof of principle. Mice knocked-out for specific genes are usually backcrossed into strains to enhance their phenotypes. Therefore, accurate characterization of phenotypical traits, such as clinical chemistry and hematological parameters, is of paramount relevance for the selection of the appropriate mouse strains for the various *in vivo* analyses, and to identify alterations more accurately versus control mice. The reference intervals for C57BL/6J mice cited in most studies refer to animals aged between 3 and 5 months [3], [4]. Reference values for the early and late phases of the life of C57BL/6J mice and for other mouse strains frequently used in animal experiments, are scanty or lacking [4], [5]. Indeed, there are differences related to the age and gender of the studied animals; moreover, blood sampling procedures differed among studies, and this is a main source of variability in reference values [5]. The aim of this study was to calculate the reference values of 5 hematological parameters and of 26 analytes relative to electrolyte balance and lipoprotein metabolism, and to muscle/heart, liver, kidney and pancreas functions in C57BL/6J, 129SV/EV and C3H/HeJ mice (30/strain) at three age ranges: 1–2 months, 3–8 months and 9–12 months. We also calculated gender-reference values for each mouse strain. The reference ranges of most analytes differed significantly in relation to the gender and age of animals. Consequently, it appears that specific reference values should be used in studies involving animal models.

## Materials and Methods

### Animals

Ninety C57BL/6J, 129SV/EV and C3H/HeJ mice (30 animals/strain: 15 male and 15 female) were examined. Animals (21 days old) were obtained from Charles River Laboratory (Wilmington, MA). They were housed in humidity-controlled (40/60%) and temperature-controlled (21±2°C) rooms with a 12-h light-cycle and separated from other animals. They had free access to water and chow (Mucedola Standard diet 4RF21, Settimo Milanese, Italy). The mice were weighed monthly. Whole blood was sampled from the retro-orbital sinus after isoflurane anaesthesia through an uncoated microhematocrit tube (Becton-Dickinson, Franklin, NJ) (previously soaked in EDTA) directly in Microtainer tubes (Becton-Dickinson) with (for hematology) and without (for biochemical assays) EDTA. Blood was collected each month from July 2005 to June 2006. On months 1, 3, 5, 7, 9 and 11, we collected 150 µL of whole blood per animal to test hematological parameters for a total of 30 samples/strain/month (540 samples in 1 year). On months 2, 4, 6, 8, 10 and 12 we collected 200 µL of blood per animal and pooled samples from 3 mice in a final volume of 600 µL to measure biochemical parameters for a total of 10 pooled samples/strain/month (180 pooled samples in 1 year). To minimize intra-individual variability we numbered animals and always pooled samples from the same animals. At the end of the experiment, all mice were killed according to approved procedures (cervical dislocation). The study was conducted in accordance with guidelines in the *Guide for the Care and Use of Laboratory Animals* [6], and the experimental protocol was approved by the Ethics Committee on Animal Experimentation of the University of Naples Federico II, Naples, Italy.

### Blood and serum analysis

Whole blood was immediately analyzed for complete blood count, i.e., red blood cell (RBC), haemoglobin (HGB), hematocrit (HCT), white blood cell (WBC) and platelets (PLT), using the fully automated ABX Pentra 60C+ Analyzer (Horiba ABX, Montpellier, France). Briefly, 53 µL of blood were aspired into a needle, divided and distributed to the various chambers for sample analysis. Pooled blood samples were centrifuged at 2500 rpm for 10 min to obtain sera, which were immediately frozen. All biochemical serum evaluations, which are used to investigate main metabolic pathways and organ functions, were performed at the same time to minimize analytical variability, and determined by dry chemistry on a Vitros 250 Analyzer (Ortho Clinical Diagnostics, Johnson & Johnson Co, Rochester, NY). We evaluated: (1) the pancreas panel: glucose (GLU), amylase (AMS) and lipase (LPS); (2) the lipid panel: triacylglycerols (TAG) and cholesterol (Chol); (3) the muscle/heart panel: lactate dehydrogenase (LDH) and total creatine kinase (CK); (4) the kidney panel: creatinine (Crea), urea (BUN) and uric acid (UA); (5) the liver panel: γ-glutamyl transferase (GGT), aspartate transaminase (AST), alanine transaminase (ALT), alkaline phosphatase (ALP), total bilirubin (t-Bil), conjugated bilirubin (c-Bil), cholinesterase (CHE), total proteins (TP), albumin (Alb) and C-reactive protein (CRP); and (6) the electrolyte panel: sodium (Na^+^), potassium (K^+^), chloride (Cl^−^), calcium (Ca^++^), magnesium (Mg^++^), phosphate (PO_4_^−^) and iron (Fe^++^).

For quality control, we used a human pooled serum and a commercial control serum (Ortho Clinical Diagnostics) to investigate imprecision and inaccuracy of the biochemical measurements. A commercial control blood (Horiba ABX) was invariably tested together with animal blood samples to test variability of hematological investigations. The intra assay variability (CV%) of biochemical assays was relative to 15 repeated determinations of the control serum in the same analytical session, whereas inter-assay CV% for each parameter was calculated on the mean values of control sera measured in 6 analytical sessions. The level of haemolysis was evaluated in randomly selected serum samples from each set of blood samples by measuring the level of haemoglobin spectrophotometrically. When necessary, serum samples were diluted with bovine serum albumin (or saline solution for Alb and TP) according to the manufacturers' indications.

### Statistic analysis

Statistic analyses were performed with SPSS v15.00. For each mouse strain, we calculated the mean and standard error of weights measured monthly. Because hematological and biochemical data had a nonparametric distribution, we report analytical results as median values and percentile intervals (2.5^th^ and 97.5^th^ percentiles). We used the Mann-Whitney and Kruskal-Wallis tests for between-group comparisons. Differences were considered statistically significant at a *p*<0.05.

## Results

**Figure 1:**
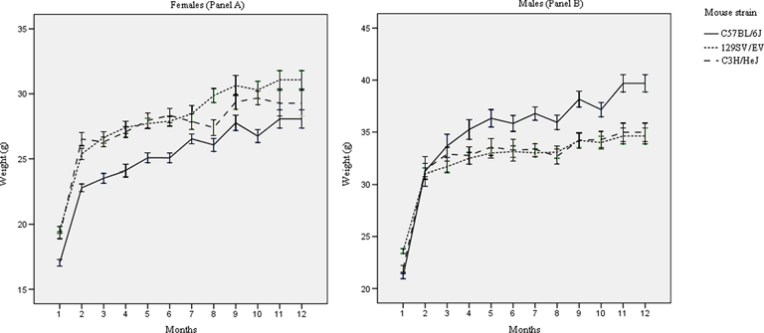
Female (Panel A) and male (Panel B) mice weight curves. Weight curves (mean±SE) of the three mouse strains (C57BL/6J, 129SV/EV and C3H/HeJ) in the one year period of the study.

Intra- and inter-assay CV% of analytical assays are reported in Table S1. Inter-assay CV% were always <7% for the hematological parameters and <10% for all biochemical analytes except t-Bil (CV = 12.22%) and CRP (CV = 10.61%). The female (panel A) and male (panel B) weight curves (mean±SE) of the three mouse strains are shown in Figure 1, and indicate that all animals grew as expected (according to weight curves from Charles River Laboratory); repeated blood collection did not cause any untoward effects.

**Figure 2:**
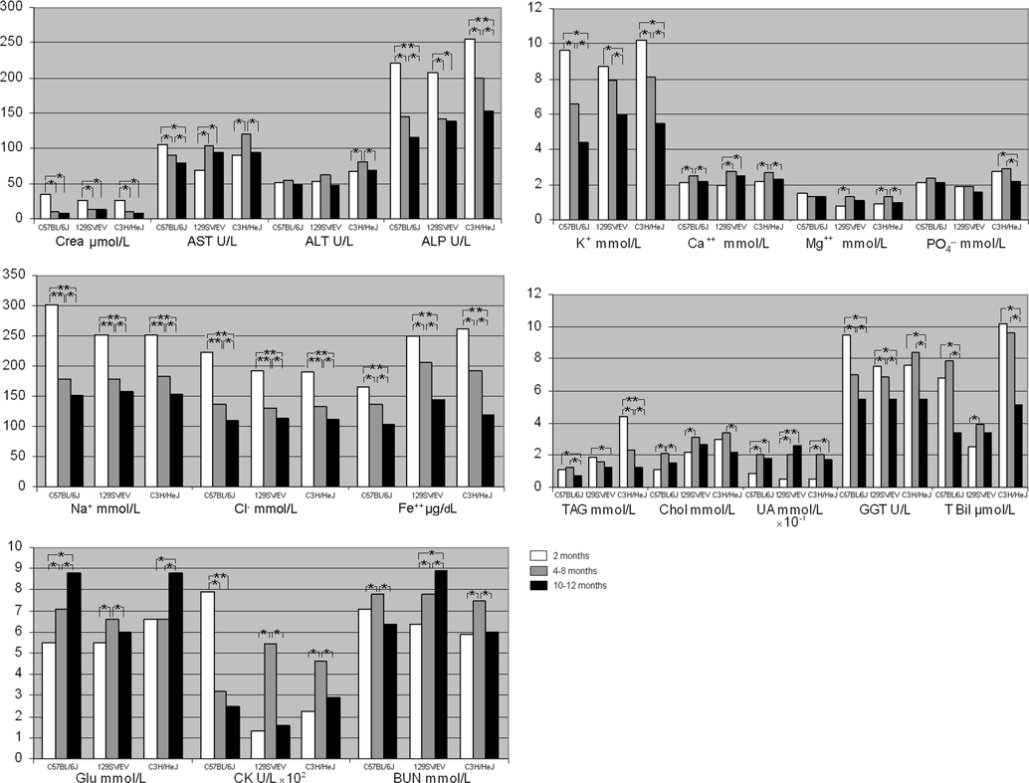
Biochemical median values in female mice. Age related biochemical median values calculated in C57BL/6J, 129SV/EV and C3H/HeJ mouse strains. Statistical significant differences among values observed in the three age-period (2/ 4–8/ 10–12 months) are also indicated: * *p*<0.05, ** *p*<0.001.

**Figure 3:**
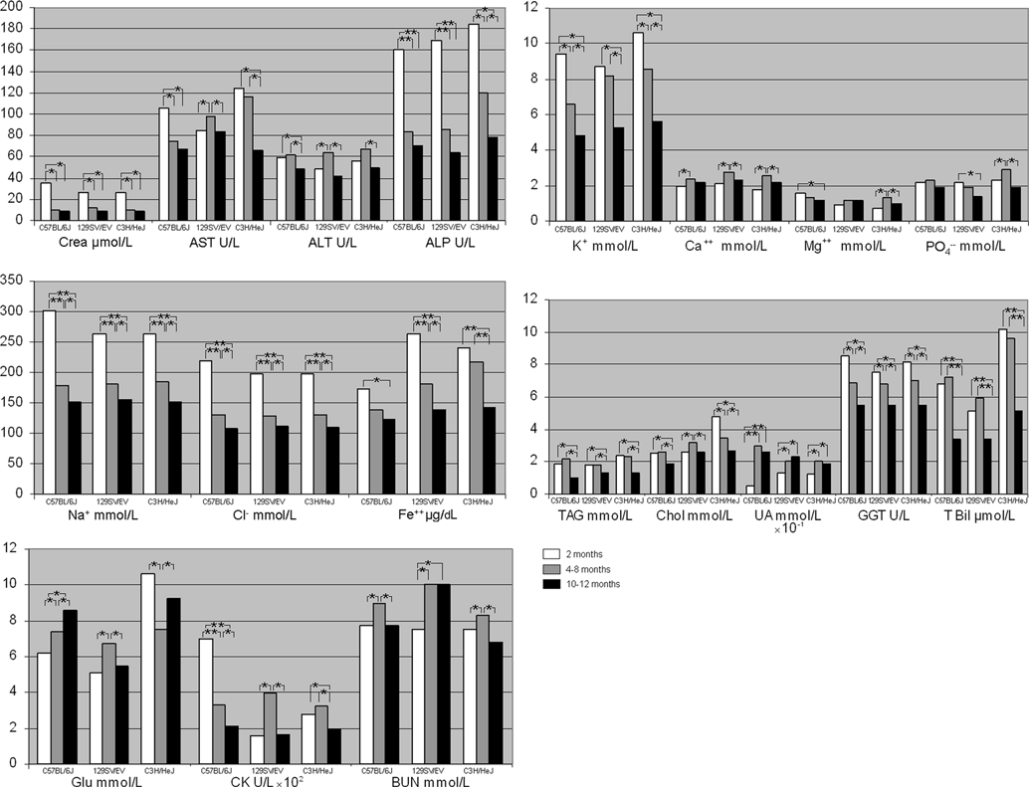
Biochemical median values in male mice. Age related biochemical median values calculated in C57BL/6J, 129SV/EV and C3H/HeJ mouse strains. Statistical significant differences among values observed in the three age-period (2/ 4–8/ 10–12 months) are also indicated: * *p*<0.05, ** *p*<0.001.

To explore age-related metabolic modifications, we measured biochemical and hematological parameters in the three mouse strains on alternate months for one year. Age-related reference intervals (median and 2.5^th^–97.5^th^ percentiles ranges) for biochemical analytes are shown in Tables 1, S2 and S3 (4–8-month, 2-month and 10–12-month-old animals, respectively). Tables 2 and S4 show the age-related reference intervals for hematological parameters in 3–7-month, and 1-month-old/9–11-month-old animals, respectively. There were both gender and inter-strain differences in biochemical analytes in animals aged between 4 and 8 months (Table 1). Specifically, in C57BL/6J mice, median levels of GLU, Chol and BUN were higher, and LPS, AST, ALP and CHE were lower in males than in females (*p* range: 0.05–0.001). In 129SV/EV mice, BUN was higher and CHE lower in males than in females (*p* range: 0.05–0.001). In C3H/HeJ mice, GLU, LDH and BUN were higher and ALP and CHE lower in males than in females (*p* range: 0.05–0.001). Similar gender dimorphisms occurred throughout the 12 months of the study (Table S2 and S3) in all mouse strains. Age-dependent variations were also recorded for most biochemical parameters in three age-ranges (Figures 2 and 3). In general, in animals 2 months old we observed the highest values of electrolytes (Na^+^, K^+^, Cl^−^, Fe^++^, *p*<0.001) and ALP activity than in the other two examined age periods (Figures 2 and 3).

**Table 1:** Serum biochemical analytes (median and 2.5^th^–97.5^th^ percentiles interval) measured in aged 4–8 months C57BL/6J, 129SV/EV and C3H/HeJ mouse strains (n = 90).

Analyte	Mouse Strain						Inter strain differences^a^
	C57BL/6J		129SV/EV		C3H/HeJ		
	Male	Female	Male	Female	Male	Female	
GLU mmol/L	7.4 b	7.1	6.7	6.6	7.5 b	6.6	129SV/EV *p*<0.05
	5.6–9.1	5.2–12.2	4.1–9.9	4.7–8.4	6.3–8.6	5.6–8.3	
LPS U/L	901	1177 b	991	1090	969	1057	
	649–1646	788–1624	689–1201	777–1419	536–1372	634–1541	
TAG mmol/L	2.2 c	1.2	1.8	1.6	2.3	2.3	C3H/HeJ *p*<0.001
	1.1–2.9	0.6–1.8	0.9–4.5	0.7–4.8	1.7–6.0	1.0–3.8	
Chol mmol/L	2.6 b	2.1	3.2	3.1	3.5	3.4	C3H/HeJ *p*<0.001
	1.8–3.9	1.3–3.4	2.2–5.3	2.1–4.1	2.8–5.2	2.2–3.6	
LDH U/L	1888	1837	2430	2240		2067	
	1590–2610	843–3150	2212–3150	1752–3150	>2250 b ** d	1829–2250	
CK U/L	327	319	398	544	324	464	
	209–635	105–649	138–615	199–964	206–660	204–921	
Crea µmol/L	10.2	10.2	12.4	12.4	10.2	10.2	
	8.8–13.2	8.8–13.2	8.8–20.5	8.8–20.5	8.8–12.3	8.8–12.4	
BUN mmol/L	9.0 b	7.8	10.0 b	7.8	8.3 b	7.5	
	7.8–11.5	6.8–10.0	6.8–13.2	6.5–10.3	7.4–12.9	6.6–10.0	
UA mmol/L	0.3	0.2	0.2	0.2	0.2	0.2	
	0.1–0.6	0.1–0.7	0.1–0.3	0.1–0.3	0.1–0.6	0.1–0.5	
GGT U/L	6.9	7	6.8	6.9	7	8.4	
	6–8	6–8	6–8	6–8	6–8	6–10	
AST U/L	75	91 b	98	104	116	121	C57BL/6J *p*<0.001
	55–91	51–122	71–201	69–194	67–160	80–172	
ALT U/L	61	55	64	63	67	80	
	46–70	42–73	45–84	46–114	39–115	56–107	
ALP U/L	84	145 c	86	142 b	120	200 c	
	67–128	103–217	68–179	97–287	75–137	126–240	
t-Bil µmol/L	7.2	7.9	5.9	3.9	9.6	9.6	129SV/EV *p*<0.05
	5.1–11.9	3.4–14.3	1.7–19.1	1.7–14.3	3.4–11.9	3.4–13.9	
c- Bil µmol/L e	0–1.7	0–5.1	0–5.1	0–3.4	0–6.8	0–6.8	
CHE U/L	4450	6880 c	5540	7550 c	5350	6790 b	
	3870–6130	5660–9770	4440–6830	5870–8620	3690–9790	5420–9560	
TP g/L	63	66	61	57	62	53	
	47–72	45–83	43–65	48–68	49–74	25–71	
Alb g/L	33	34	29	27	31	31	
	22–42	20–47	18–31	20–36	22–39	24–41	
CRP mg/L e	0–0.7	0–0.7	0–2.8	0–1.4	0–0.7	0–2.3	
Na^+^ mmol/L	179.2	179.8	180.9	178.6	184.4	182.1	
	151.0–254.8	149.0–281.4	147.0–253.4	148.0–253.4	151.0–247.8	150.0–245.0	
K^+^ mmol/L	6.6	6.6	8.2	7.9	8.6	8.1	C57BL/6J *p*<0.05
	5.2–14.5	4.0–14.0	4.9–10.3	5.0–9.5	5.8–13.1	6.0–13.4	
Cl^−^ mmol/L	131.1	137.4	128.7	131.1	131.1	132.2	
	109.0–179.2	110.0–204.4	106.0–179.2	110.0–184.8	110.0–172.2	110.0–177.8	
Ca^++^ mmol/L	2.4	2.5	2.8	2.8	2.6	2.7	C57BL/6J *p*<0.05
	2.2–2.6	2.3–3.5	2.3–3.4	2.3–3.0	2.2–2.9	2.3–2.9	
Mg^++^ mmol/L	1.3	1.3	1.2	1.3	1.3	1.3	
	1.1–1.7	1.2–1.8	1.0–1.4	0.9–1.4	0.9–1.8	1.0–1.9	
PO_4_^−^ mmol/L	2.3	2.4	1.9	1.9	2.9	2.9	129SV/EV *p*<0.05
	2.1–2.7	2.0–3.1	1.4–3.0	1.5–3.1	1.6–3.6	1.8–3.5	
Fe^++^µg/dL	138.0	136.8	181.5	206.5	216.9	191.4	C57BL/6J *p*<0.001
	102.0–190.4	70.0–184.8	121.0–273.0	111.0–280.7	142.0–334.6	131.0–320.6	

a Significantly different values in the reported mouse strain vs the other strains.

b c Significantly different intersex mouse strain values: *p*<0.05 and *p*<0.001 respectively.

d Analyte value above the upper linearity level of the method on undiluted sample.

e min-max values.

**Table 2:** Hematological parameters (median and 2.5^th^–97.5^th^ percentiles interval) measured in aged 3–7 months C57BL/6J, 129SV/EV and C3H/HeJ mouse strains (n = 90).

Mouse Strains	Sex	Parameters				
		WBC (10^3^/mm^3^)	RBC (10^6^/mm^3^)	HGB (g/dL)	HCT (%)	PLT (10^3^/mm^3^)
C57BL/6J	M	8.20 a	8.97	13.10	41.50	754.50
		2.47–14.42	2.93–11.01	4.10–16.80	12.50–53.00	155.55–1036.88
	F	6.55	9.56 b	14.30 b	45.65 b	756.50
		2.20–11.53	3.47–11.73	5.70–17.00	16.20–58.30	144.00–894.00
129SV/EV	M	8.10	7.97	12.40	37.90	464.50 b
		3.86–11.67	3.42–11.40	6.20–17.70	15.90–54.60	255.00–612.00
	F	8.30	8.87	13.80	42.35	389.00
		5.90–10.10	4.94–11.11	5.16–17.38	15.09–53.35	170.10–538.30
C3H/HeJ	M	8.20 a	7.69	12.40	37.30	647.00 b
		5.50–11.60	4.22–9.93	6.70–16.00	20.10–49.10	344.00–960.00
	F	6.40	7.48	12.10	36.20	521.00
		2.80–10.80	3.80–10.19	6.30–16.30	18.00–50.50	115.00–840.00
Inter strain differences c			C3H/HeJ *p*<0.05	C3H/HeJ *p*<0.05	C3H/HeJ *p*<0.05	C57BL/6J *p*<0.05

a b Statistically significant intersex mouse strain different values: *p*<0.05 and *p*<0.001 respectively.

c Statistically significant different values in the reported mouse strain vs the other strains. M: male; F: female.

AMS values in all strains and in all age-ranges, and LDH activity in some instances (in 4–8-month old C3H/HeJ mice, and in 10–12-month old 129SV/EV mice) were above the upper linearity level of the method, and we were unable to repeat the tests due to scarcity of biological samples.

**Figure 4:**
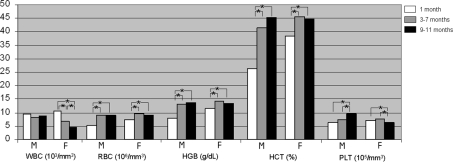
Hematological median values in female and male mice. Age related hematological median values calculated in C57BL/6J mouse strain (M = males; F = females). Statistical significant differences among values observed in the three age-period (1/ 3–7/ 9–11 months) are also indicated: * *p*<0.05.

We evaluated haemolysis caused by retro-orbital blood sampling in randomly selected animals in each set of blood collections; haemolysis was moderate, i.e., <0.7 g/dL. Significant inter-strain differences were observed for GLU, t-Bil, K^+^, Ca^++^ and PO_4_^−^ (*p*<0.05), and for TAG, Chol, AST and Fe^++^ (*p*<0.001) in the 4–8-month-old animals (Table 1). Inter-strain differences (*p* range: 0.05–0.001) were observed for CK, Crea, BUN, Na^++^, K^+^, Cl^−^ and Mg^++^ among the animals aged 2 months and/or 10–12 months (Tables S2 and S3). C57BL/6J females aged between 3 and 7 months had slightly higher RBC, HGB and HCT values and lower WBC values than their male counterparts (*p* range: 0.05–0.001) (Table 2). In the same age range, gender differences were observed in 129SV/EV and in C3H/HeJ animals as regards PLT values (*p*<0.001), and WBC and PLT values (*p* range: 0.05–0.001), respectively. Further, inter-strain differences were recorded for RBC, HGB, HCT and PLT values (*p*<0.05, Table 2) in mice aged between 3 and 7 months. Similar hematological parameters differences between sexes and strains emerged in 1-month-old and in 9–11-month-old animals (Table S4). Moreover, RBC, HGB, HCT and PLT, but not WBC, tended to increase during the 1-year life span of C57BL/6J mice (Figure 4). Hematological parameters were unrelated to age in the 129SV/EV and C3H/HeJ male mice (data not shown).

## Discussion

In this study, we used a standardized retro-orbital procedure of blood collection, and calculated reference intervals for numerous biochemical and hematological parameters in 90 animals of the mouse strains C57BL/6J, 129SV/EV and C3H/HeJ in three periods of a 1-year life span.

To our knowledge, this is the first report of: (1) biochemical and hematological reference ranges in 129SV/EV mice; (2) biochemical reference ranges in C3H/HeJ mice, and (3) reference values of GGT, LPS, CHE, CRP, Cl^−^, Mg^++^, and Fe^++^ parameters. Therefore, our data could improve the evaluation of changes of metabolism in these experimental animals versus control animals.

Compared with previous reports, our data on C57BL/6J mice aged 4–8 months showed:

1. Lower median GLU values in both females and males, namely 7.1 mmol/L and 7.4 mmol/L, respectively versus 8.3 mmol/L and 11.4 mmol/L [3], [4], [7], [8]. The discrepancy could be due to less stressing housing conditions of our animals.
2. Comparable median values for TAG in females (1.2 mmol/L), but higher in males (2.2 mmol/L) than those previously reported (female/male range: 0.9–1.3 mmol/L) [3], [4], [8]. This discrepancy could depend both on different feeding and ages of investigated animals.
3. Comparable median values of Chol, TP, Alb, Ca^++^, BUN, t-Bil, UA, ALT and ALP activities to previously reported values [3]–[5], [7], [8].
4. Higher levels of K^+^, (6.6 mmol/L), AST (75 M and 91 F U/L), LDH (1888 M and 1837 F U/L) than those previously obtained with intracardiac blood collection (respectively: 4.3–6.4 mmol/L K^+^; 45–68 U/L AST; 293–348 U/L LDH) [3], [4], [7], [8]. Haemolysis consequent to retro-orbital blood sampling could explain these contrasting data. However, haemolysis in our samples was moderate, in fact our above reported AST levels were lower than those previously described after retro-orbital blood sampling (209–255 U/L AST) [5].
5. Similar CK median results for females (319 U/L versus F 358 U/L) and lower for males (327 U/L versus M 601 U/L) to those in the Mouse Phenome Database [8]. Differences in CK values (from 102 to 2000 U/L) were previously reported [3], [5], this could depend on different blood collection technique used.

Regarding hematological parameters in both C57BL/6J and C3H/HeJ mice aged 3–7 months, we obtained higher WBC median values in males (8.20×10^3^/mm^3^) than in females (6.55 and 6.40×10^3^/mm^3^) in according with other authors, blood sampling technique being equal [5], [9], namely 6.2–6.6×10^3^/mm^3^ in males and 4.6–5.9×10^3^/mm^3^ in females. In addition, our RBC, HGB, HCT and PLT median values were about 10% lower than those reported by others [5], [7]–[9]. Repeated blood sample collections could explain the slight difference between these studies.

Most of the biochemical analytes we examined differed according to age in all three mouse strains. This finding supports the use of age-specific reference intervals for biochemical analytes in studies involving these animals. Concerning hematological parameters, RBC, HGB, HCT and PLT tended to increase with the animal's age only in C57BL/6J mice. In addition, inter-strain differences were observed in all three age periods for most of the tested parameters. This supports the use of strain-specific reference values.

In conclusion, we suggest that strain-specific gender- and age-dependent reference intervals for biochemical and hematological parameters be used to evaluate differences between experimental animals and controls. In fact, these reference intervals are tools that can lead to a more precise evaluation of changes after therapeutic intervention in animal models of human diseases. In addition, precise reference intervals for biochemical and hematological parameters will allow a more detailed characterization of mouse mutants harboring mutations that often do not induce striking phenotypes, such as micro-RNAs depletion.

## Supporting Information

Table S1 Intra- and inter-assay coefficients of variation (CV) for biochemical and haematological analytes evaluated respectively in a control serum with the Vitros 250 Chemistry System analyzer (Ortho-Clinical Diagnostics) and in a control blood sample obtained from Pentra C60+ (Horiba ABX).
(0.05 MB DOC)

Table S2 Serum biochemical analytes (median and 2.5th–97.5th percentiles interval) measured in aged 2 months C57BL/6J, 129SV/EV and C3H/HeJ mouse strains (n = 90).
(0.12 MB DOC)

Table S3 Serum biochemical analytes (median and 2.5th–97.5th percentiles interval) measured in aged 10–12 months C57BL/6J, 129SV/EV and C3H/HeJ mouse strains (n = 90).
(0.12 MB DOC)

Table S4 Hematological parameters (median and 2.5th–97.5th percentiles interval) measured in aged 1 month and 9–11 months C57BL/6J, 129SV/EV and C3H/HeJ mouse strains (n = 90).
(0.06 MB DOC)
