# Supplementary material for: Age-Related Reference Intervals of the Main Biochemical and Hematological Parameters in C57BL/6J, 129SV/EV and C3H/HeJ Mouse Strains
Source: PLoS One. 2008 Nov 20;3(11):e3772. doi: 10.1371/journal.pone.0003772 (PMC2582346; doi:10.1371/journal.pone.0003772)
Supplement: Table S1 — Intra- and inter-assay coefficients of variation (CV) for biochemical and haematological analytes evaluated respectively in a control serum with the Vitros 250 Chemistry System analyzer (Ortho-Clinical Diagnostics) and in a control blood sample obtained from Pentra C60+ (Horiba ABX). (0.05 MB DOC) [file pone.0003772.s001.doc]

Table S1: Intra- and inter-assay coefficients of variation (CV) for biochemical and haematological analytes evaluated respectively in a control serum with the Vitros 250 **Chemistry System** analyzer (Ortho-Clinical Diagnostics) and in a control blood sample obtained from Pentra C60+ (Horiba ABX).

| **Analyte *^a^***  **(mean control value)** | **GLU**  **(5.54 mmol/L)** | **LPS**  **(71 U/L)** | **TAG**  **(1.60 mmol/L)** | **Chol**  **(4.20 mmol/L)** | **LDH**  **(718 U/L)** | **CK**  **(92 U/L)** | **Crea**  **(84.86 µmol/L)** | **BUN**  **(7.01 mmol/L)** |
| --- | --- | --- | --- | --- | --- | --- | --- | --- |
| **CV% Inter-assay *^b^*** | 3.49 | 4.89 | 2.13 | 1.84 | 2.14 | 5.19 | 5.40 | 1.98 |
| **CV% Intra-assay *^c^***  **(min-max)** | 0.89 - 1.16 | 1.19 - 2.55 | 0.67 - 1.47 | 1.10 - 2.81 | 1.23 - 1.70 | 1.66 - 4.46 | 0.00 - 4.50 | 0.00 - 2.16 |
|  | **UA**  **(0.324 mmol/L )** | **GGT**  **(52 U/L)** | **AST**  **(31 U/L)** | **ALT**  **(30 U/L)** | **ALP**  **(112 U/L)** | **t-Bil**  **(13.68 µmol/L)** | **CHE**  **(6280 U/L)** | **TP**  **(6.8 g/dL)** |
| **CV% Inter-assay *^b^*** | 4.77 | 5.58 | 7.08 | 6.55 | 6.98 | 12.22 | 2.28 | 3.41 |
| **CV% Intra-assay *^c^***  **(min-max)** | 0.75 - 1.04 | 0.82 - 1.94 | 0.00 - 1.77 | 3.41 - 6.67 | 1.24 - 2.26 | 0.00 - 5.86 | 1.26 - 2.77 | 1.06 - 1.90 |
|  | **Alb**  **(4 g/dL)** | **CRP**  **(210 mg/L)** | **Na^+^**  **(148.5 mmol/L)** | **K^+^**  **(4.4 mmol/L)** | **Cl^-^**  **(108.8 mmol/L)** | **Ca^++^**  **(2.49 mmol/L)** | **PO_4_^--^**  **(1.29 mmo/L)** | **Fe^++^**  **(70 µg/dL)** |
| **CV% Inter-assay *^b^*** | 5.02 | 10.61 | 3.59 | 2.31 | 1.73 | 5.57 | 2.67 | 7.64 |
| **CV% Intra-assay *^c^***  **(min-max)** | 1.25 - 1.77 | 2.58 - 6.89 | 0.65 - 1.01 | 0.84 - 1.19 | 0.73 - 1.08 | 0.93 - 1.55 | 0.97 - 1.25 | 1.41 - 3.27 |
|  | **WBC**  **(10.25 10^3^/mm^3^)** | **RBC**  **(4.59 10^6^/ mm^3^)** | **HGB**  **(13.42 g/dL)** | **HCT**  **(37.38 %)** | **PLT**  **(251.17 10^3^/mm^3^)** |  |  |  |
| **CV% Inter-assay *^b^*** | 5.13 | 3.36 | 1.52 | 2.93 | 6.95 |  |  |  |
| **CV% Intra-assay *^c^***  **(min-max)** | 0.67-1.45 | 0.29-1.59 | 0.53-1.06 | 0.19-2.18 | 0.26-4.80 |  |  |  |

***^a^*** GLU: Glucose, LPS: Lipase, TAG: Triacylglyceroles, Chol: cholesterol, LDH: Lactate dehydrogenase, CK: Creatine kinase, Crea: Creatinine, BUN: Blood urea nitrogen, UA: Uric acid, GGT: γ-Glutamyl-transferase, AST: Aspartate transaminase, ALT: Alanine transaminase, ALP: Alkaline phosphatase, t- Bil: total bilirubin, CHE: Cholinesterase, TP: Total proteins, Alb: Albumin, CRP: C reactive protein, WBC: White blood cells, RBC: Red blood cells, HGB: Haemoglobin, HCT: Haematocrit, PLT: Platelets.

***^b^*** Inter-assay CV% was based on mean control serum values obtained in 6 analytical sessions.

***^c^*** Min-max intra-assay CV% values of 6 analytical sessions are reported. (Intra-assay CV% was based on mean value of 15 repeated determinations of the control serum in the same analytical session for biochemical analytes and/or mean value of 2 repeated determinations of the control blood in the same analytical session for haematological parameters).
