# Supplementary material for: Age-Related Reference Intervals of the Main Biochemical and Hematological Parameters in C57BL/6J, 129SV/EV and C3H/HeJ Mouse Strains
Source: PLoS One. 2008 Nov 20;3(11):e3772. doi: 10.1371/journal.pone.0003772 (PMC2582346; doi:10.1371/journal.pone.0003772)
Supplement: Table S2 — Serum biochemical analytes (median and 2.5th–97.5th percentiles interval) measured in aged 2 months C57BL/6J, 129SV/EV and C3H/HeJ mouse strains (n = 90). (0.12 MB DOC) [file pone.0003772.s002.doc]

Table S2: Serum biochemical analytes (median and 2.5^th^-97.5^th^ percentiles interval) measured in aged 2 months C57BL/6J, 129SV/EV and C3H/HeJ mouse strains (n=90).

| Analyte*^a^* | Mouse Strain | | | | | | Inter strain differences*^b^* |
| --- | --- | --- | --- | --- | --- | --- | --- |
|  | C57BL/6J | | 129SV/EV | | C3H/HeJ | |  |
|  | M | F | M | F | M | F |  |
| GLU mmol/L | 6.2 | 5.5 | 5.1 | 5.5 | 10.6 *^c^* | 6.6 | C3H/HeJ *p*<0.05 |
|  | 4.8-6.7 | 5.1-5.9 | 4.5-5.5 | 4.4-6.2 | 7.6-14.9 | 5.9-9.9 |  |
| LPS U/L | 978 | 1174 *^c^* | 1006 | 1083 | 999 | 954 |  |
|  | 926-1074 | 1064-1254 | 876-1105 | 967-1102 | 829-1044 | 846-1360 |  |
| TAG mmol/L | 1.9 | 1.1 | 1.8 | 1.9 | 2.4 | 4.4 | C3H/HeJ *p*<0.05 |
|  | 1.1-3.4 | 0.2-1.3 | 1.6-2.0 | 1.7-2.4 | 1.6-3.2 | 2.8-4.8 |  |
| Chol mmol/L | 2.5 |  | 2.6 | 2.2 | 4.8 *^c^* | 3.0 | C3H/HeJ *p*<0.05 |
|  | 2.3-2.6 | <1.16 *^d^* | 2.1-2.7 | 2.1-2.4 | 4.0-8.2 | 2.8-4.3 |  |
| LDH U/L | 4222 | 2513 | 2220 | 2101 | 3339 | 2785 |  |
|  | 4080-4500 | 2280-2780 | 1965-2566 | 1986-2251 | 3304-3375 | 2311-3375 |  |
| CK U/L | 702 | 790 | 156 | 132 | 274 | 228 | C57BL/6J *p*<0.05 |
|  | 668-866 | 182-998 | 147-256 | 127-180 | 265-284 | 187-255 |  |
| Crea µmol/L | 35.4 | 35.4 | 26.5 | 26.5 | 26.5 | 26.5 | C57BL/6J *p*<0.05 |
|  | 26.5-44.2 | 26.5-35.3 | 25.6-27.4 | 25.6-27.4 | 22.1-39.8 | 22.1-30.9 |  |
| BUN mmol/L | 7.7 | 7.1 | 7.5 | 6.4 | 7.5 | 5.9 |  |
|  | 7.8-7.9 | 5.7-8.5 | 6.4-8.0 | 5.9-6.9 | 5.9-13.4 | 4.8-8.0 |  |
| UA mmol/L | 0.05 | 0.09 *^c^* | 0.13 *^c^* | 0.05 | 0.12 | 0.05 |  |
|  | 0.04-0.06 | 0.08-0.10 | 0.07-0.16 | 0.04-0.09 | 0.09-0.20 | 0.04-0.15 |  |
| GGT U/L | 8.5 | 9.5 | 7.5 | 7.5 | 8.2 | 7.6 |  |
|  | 8-9 | 8-10 | 6.5-8 | 7.5-8 | 7.5-9 | 7.5-10.5 |  |
| AST U/L | 106 | 105 | 85 | 70 | 124 | 91 |  |
|  | 86-110 | 66-136 | 55-112 | 61-81 | 75-174 | 79-117 |  |
| ALT U/L | 59 | 51 | 48 | 52 | 56 | 67 |  |
|  | 58-60 | 46-54 | 43-54 | 49-55 | 51-61 | 51-81 |  |
| ALP U/L | 160 | 221 | 169 | 208 *^c^* | 185 | 256 |  |
|  | 136-178 | 214-246 | 150-187 | 201-222 | 147-223 | 232-367 |  |
| t-Bil µmol/L | 6.8 | 6.8 | 5.1 | 2.5 | 10.2 | 10.2 |  |
|  | 6.8-10.2 | 3.4-6.8 | 2.5-5.1 | 2.5-5.1 | 7.7-25.6 | 7.7-15.3 |  |
| c-Bil µmol/L *^e^* | 0 - 1.71 | 0 - 1.71 | 0 - 1.71 | 0 - 1.71 | 0 - 1.71 | 0 - 1.71 |  |
| CHE U/L | 3860 | 5210 | 4200 | 4970 *^c^* | 4770 | 5010 |  |
|  | 3600-4040 | 4620-5380 | 3980-4340 | 4770-5670 | 3870-4960 | 4310-7530 |  |
| CRP mg/L *^e^* | 0-0.7 | 0-0.7 | 0-0.7 | 0-0.7 | 0-0.7 | 0-0.7 |  |
| Na^+^ mmol/L | 302 | 302 | 264 | 252 | 264 | 252 | C57BL/6J *p*<0.05 |
|  | 298-304 | 300-304 | 256-264 | 246-271 | 252-267 | 249-264 |  |
| K^+^ mmol/L | 9.4 | 9.6 | 8.7 | 8.7 | 10.6 | 10.2 |  |
|  | 9.2-9.4 | 9.2-9.6 | 8.5-9.0 | 8.6-9.0 | 10.2-12.9 | 9.9-10.5 |  |
| Cl^-^ mmol/L | 220.0 | 223.5 *^c^* | 198.0 | 192.0 | 198.0 | 190.5 | C57BL/6J *p*<0.05 |
|  | 218.0-222.0 | 220.0-224.0 | 192.0-199.5 | 187.5-205.5 | 187.5-201.0 | 189.0-198.0 |  |
| Ca^++^ mmol/L | 2.0 | 2.1 | 2.1 | 2.0 | 1.8 | 2.2 |  |
|  | 1.9-2.1 | 2.1-2.2 | 1.9-2.2 | 1.9-2.2 | 1.3-2.2 | 1.9-3.3 |  |
| Mg^++^ mmol/L | 1.6 | 1.5 | 0.9 *^c^* | 0.8 | 0.7 | 0.9 | C57BL/6J *p*<0.05 |
|  | 1.5-1.6 | 1.4-1.6 | 0.8-0.9 | 0.7-0.8 | 0.4-0.9 | 0.8-1.3 |  |
| PO_4_ -- mmol/L | 2.2 | 2.1 | 2.2 *^c^* | 1.9 | 2.3 | 2.8 |  |
|  | 1.8-2.6 | 2.1-2.5 | 1.9-2.3 | 1.6-2.0 | 1.8-2.8 | 2.5-3.7 |  |
| Fe^++^ µg/dL | 174.0 | 165.0 | 264.0 | 250.5 | 240.7 | 262.5 | C57BL/6J *p*<0.05 |
|  | 148.0-184.0 | 160.0-182.0 | 246.0-270.0 | 238.5-313.5 | 192.0-289.50 | 210.0-346.5 |  |

*^a^* GLU: Glucose, LPS: Lipase, TAG: Triacylglyceroles, Chol: Cholesterol, LDH: Lactate dehydrogenase, CK: Creatine kinase, Crea: Creatinine, BUN: Blood urea nitrogen, UA: Uric acid, GGT: γ-glutamyl-transferase, AST: Aspartate transaminase, ALT: Alanine transaminase, ALP: Alkaline phosphatase, t-Bil: total bilirubin, c-Bil: conjugated bilirubin, CHE: Cholinesterase, TP: Total proteins, Alb: Albumin, CRP: C reactive protein. Alb and TP data are not available due to scarcity of samples.

*^b^* Statistically significant different values in the reported mouse strain vs the other strains; *^c^* Statistically significant intersex mouse strain different values: *p*<0.05; *^d^* below the linearity level; *^e^* min-max values.

*^a^* GLU: glucose, LPS: lipase, TAG: triacylglyceroles, Chol: cholesterol, LDH: lactate dehydrogenase, CK: creatine kinase, Crea: creatinine, BUN: blood urea nitrogen, UA: uric acid, GGT: γglutamyl-transferase, AST: aspartate transaminase, ALT: alanine transaminase, ALP: alkaline phosphatase, t-Bil: total bilirubin, c-Bil: conjugated bilirubin, CHE: cholinesterase, TP: total proteins, Alb: albumin, CRP: C reactive protein.

*^b^* Statistically significant different values in the reported mouse strain vs the other strains; *^c^* *^d^* Statistically significant intersex mouse strain different values: *p*<0.05 and *p*<0.001 respectively; *^e^* above upper linearity level of the method on undiluted sample; *^f^* min-max values.
