# Supplementary material for: Age-Related Reference Intervals of the Main Biochemical and Hematological Parameters in C57BL/6J, 129SV/EV and C3H/HeJ Mouse Strains
Source: PLoS One. 2008 Nov 20;3(11):e3772. doi: 10.1371/journal.pone.0003772 (PMC2582346; doi:10.1371/journal.pone.0003772)
Supplement: Table S3 — Serum biochemical analytes (median and 2.5th–97.5th percentiles interval) measured in aged 10–12 months C57BL/6J, 129SV/EV and C3H/HeJ mouse strains (n = 90). (0.12 MB DOC) [file pone.0003772.s003.doc]

Table S3: Serum biochemical analytes (median and 2.5^th^-97.5^th^ percentiles interval) measured in aged 10-12 months C57BL/6J, 129SV/EV and C3H/HeJ mouse strains (n=90).

| Analyte*^a^* | Mouse Strains | | | | | | Inter strain differences*^b^* |
| --- | --- | --- | --- | --- | --- | --- | --- |
|  | C57BL/6J | | 129SV/EV | | C3H/HeJ | |  |
|  | M | F | M | F | M | F |  |
| GLU mmol/L | 8.6 | 8.8 | 5.5 | 6.0 | 9.2 | 8.8 | 129SV/EV  *p*<0.001 |
|  | 6.3-10.5 | 6.2-11.6 | 4.5-7.6 | 4.9-7.2 | 7.6-10.5 | 7.0-11.6 |  |
| LPS U/L | 692 | 924 *^d^* | 814 | 835 | 673 | 786 |  |
|  | 609-754 | 868-978 | 690-986 | 539-903 | 586-754 | 583-987 |  |
| TAG mmol/L | 1.0 *^d^* | 0.7 | 1.3 | 1.2 | 1.3 | 1.2 |  |
|  | 0.8-1.2 | 0.5-0.9 | 0.8-1.8 | 0.9-1.6 | 0.8-2.2 | 0.5-2.7 |  |
| Chol mmol/L | 1.9 | 1.5 | 2.6 | 2.7 | 2.7 | 2.2 | C57BL/6J *p*<0.05 |
|  | 1.6-2.3 | 1.3-1.8 | 2.2-3.1 | 2.2-3.3 | 1.7-3.8 | 1.4-2.9 |  |
| LDH U/L | 2250 *^c^* | 1609 |  | 2250 | 2250 | 2201 |  |
|  | 1947-2250 | 1101-2250 | >2250 *^e^* | 1944-2762 | 1692-2250 | 1101-2250 |  |
| CK U/L | 214 | 246 | 163 | 160 | 190 | 291 *^c^* | 129SV/EV  *p*<0.05 |
|  | 101-468 | 144-493 | 97-414 | 122-445 | 101-452 | 144-809 |  |
| Crea µmol/L | 8.9 | 8.8 | 8.8 | 13.2 | 8.8 | 8.8 |  |
|  | 8.8-17.7 | 7.9-9.7 | 7.9-9.7 | 8.8-17.6 | 5.3-13.2 | 6.2-13.2 |  |
| BUN mmol/L | 7.7 *^d^* | 6.4 | 10.0 *^c^* | 8.9 | 6.8 *^c^* | 6.0 | 129SV/EV  *p*<0.001 |
|  | 7.1-8.9 | 5.7-7.8 | 7.8-11.8 | 6.8-10.7 | 5.3-8.5 | 5.3-6.4 |  |
| UA mmol/L | 0.26 | 0.18 | 0.23 | 0.26 | 0.19 | 0.17 |  |
|  | 0.15-0.40 | 0.06-0.30 | 0.17-0.32 | 0.17-0.34 | 0.15-0.26 | 0.06-0.24 |  |
| GGT U/L *^f^* | 5-6 | 5-6 | 5-6 | 5-6 | 5-6 | 5-6 |  |
| AST U/L | 67 | 79 | 84 | 94 | 66 | 94 *^c^* |  |
|  | 56 -104 | 66-120 | 70-92 | 56-92 | 56-105 | 66-130 |  |
| ALT U/L | 48 | 50 | 42 | 47 | 49 | 70 *^c^* |  |
|  | 39-65 | 45-58 | 37-58 | 36-54 | 44-91 | 45 -114 |  |
| ALP U/L | 70 | 116 *^d^* | 64 | 138 *^d^* | 78 | 153 *^d^* |  |
|  | 60-85 | 80-155 | 55-78 | 67-164 | 62-111 | 80-185 |  |
| t-Bil µmol/L | 3.4 | 3.4 | 3.4 | 3.4 | 5.1 | 5.1 |  |
|  | 1.7-5.1 | 1.7-5.1 | 1.7-5.1 | 1.7-6.8 | 3.4-8.5 | 3.4-8.5 |  |
| c-Bil µmol/L *^f^* | 0 - 1.71 | 0 - 3.42 | 0 - 5.13 | 0 - 3.42 | 0 - 1.71 | 0 - 5.13 |  |
| CHE U/L | 4240 | 5610 *^d^* | 4710 | 6610 *^d^* | 4210 | 5450 *^d^* |  |
|  | 4000-4410 | 5120-5960 | 4130-5030 | 4280-7980 | 4000-4450 | 5110-5700 |  |
| TP g/L | 58 | 57 | 52 | 59 | 59 | 56 |  |
|  | 57-60 | 54-59 | 47-56 | 49-97 | 52-60 | 51-59 |  |
| Alb g/L | 24 | 24 | 22 | 28 *^d^* | 25 | 26 |  |
|  | 23-25 | 22-25 | 21-26 | 24-52 | 24-27 | 22-31 |  |
| CRP mg/L *^f^* | 0-3 | 0-4 | 0-1 | 0-5 | 0-4 | 0-4 |  |
| Na^+^ mmol/L | 152.5 | 151.5 | 155.5 | 157.0 | 152.5 | 153.0 |  |
|  | 150.0-154.0 | 147.0-153.0 | 153.0-160.0 | 153.0-175.0 | 150.0-155.0 | 147.0-155.0 |  |
| K^+^ mmol/L | 4.8 *^d^* | 4.4 | 5.3 | 6.0 *^c^* | 5.6 | 5.5 | C57BL/6J *p*<0.05 |
|  | 4.5-5.3 | 4.0-4.8 | 5.0-6.0 | 5.4-6.6 | 4.5-6.7 | 4.4-7.6 |  |
| Cl^-^ mmol/L | 107.0 | 109.0 *^d^* | 111.0 | 113.0 *^c^* | 109.5 | 111.0 |  |
|  | 105.0-108.0 | 107.0-111.0 | 106.0-115.0 | 112.0-124.0 | 107.0-113.0 | 107.0-114.0 |  |
| Ca^++^ mmol/L | 2.2 | 2.2 | 2.3 | 2.5 *^d^* | 2.2 | 2.3 |  |
|  | 2.1-2.3 | 2.1-2.3 | 2.2-2.5 | 2.4-2.7 | 2.1-2.3 | 2.2-2.6 |  |
| Mg^++^ mmol/L | 1.2 | 1.3 | 1.2 | 1.1 | 1.0 | 1.0 |  |
|  | 1.0-1.4 | 1.1-1.4 | 1.1-1.3 | 1.0-1.5 | 0.9-1.2 | 0.9-1.2 |  |
| PO_4_ -- mmol/L | 1.9 | 2.1 *^c^* | 1.4 | 1.6 | 1.9 | 2.2 *^d^* | 129SV/EV  *p*<0.05 |
|  | 1.8-2.1 | 1.9-2.2 | 1.2-1.6 | 1.2-1.9 | 1.8-2.0 | 2.0-2.4 |  |
| Fe^++^ µg/dL | 124.0 *^d^* | 104.0 | 138.5 | 145.0 | 141.5 | 118.5 |  |
|  | 105.0-148.0 | 88.0-112.0 | 100.0-158.0 | 119.0-201.0 | 115.0-182.0 | 88.0-179.0 |  |

*^a^* GLU: Glucose, LPS: Lipase, TAG: Triacylglyceroles, Chol: Cholesterol, LDH: Lactate dehydrogenase, CK: Creatine kinase, Crea: Creatinine, BUN: Blood urea nitrogen, UA: Uric acid, GGT: γ-glutamyl-transferase, AST: Aspartate transaminase, ALT: Alanine transaminase, ALP: Alkaline phosphatase, t-Bil: total bilirubin, c-Bil: conjugated bilirubin, CHE: Cholinesterase, TP: Total proteins, Alb: Albumin, CRP: C reactive protein.

*^b^* Statistically significant different values in the reported mouse strain vs the other strains; *^c^* *^d^* Statistically significant intersex mouse strain different values: *p*<0.05 and *p*<0.001 respectively; *^e^* above upper linearity level of the method on undiluted sample; *^f^* min-max values.
