# Supplementary material for: Age-Related Reference Intervals of the Main Biochemical and Hematological Parameters in C57BL/6J, 129SV/EV and C3H/HeJ Mouse Strains
Source: PLoS One. 2008 Nov 20;3(11):e3772. doi: 10.1371/journal.pone.0003772 (PMC2582346; doi:10.1371/journal.pone.0003772)
Supplement: Table S4 — Hematological parameters (median and 2.5th–97.5th percentiles interval) measured in aged 1 month and 9–11 months C57BL/6J, 129SV/EV and C3H/HeJ mouse strains (n = 90). (0.06 MB DOC) [file pone.0003772.s004.doc]

Table S4: Hematological parameters (median and 2.5^th^-97.5^th^ percentiles interval) measured in aged 1 month and 9-11 months C57BL/6J, 129SV/EV and C3H/HeJ mouse strains (n=90).

| Age Range | Strain | Sex | Parameters  *^a^* | | | | |
| --- | --- | --- | --- | --- | --- | --- | --- |
|  |  |  | WBC (10^3^/mm^3^) | RBC (10^6^/ mm^3^) | HGB (g/dL) | HCT (%) | PLT (10^3^/mm^3^) |
| 1 month  (F=15) (M=15) | C57BL/6J | M | 9.60 | 5.27 | 8.10 | 26.30 | 639.00 |
|  |  |  | 7.00-11.60 | 2.36-10.12 | 3.80-15.80 | 11.60-52.00 | 263.00-971.00 |
|  |  | F | 10.70 | 7.35 *^b^* | 11.60 | 38.50 *^b^* | 716.00 |
|  |  |  | 7.10-12.20 | 2.40-9.62 | 3.80-15.30 | 11.90-50.40 | 295.00-839.00 |
|  | 129SV/EV | M | 10.15 | 6.70 *^b^* | 11.70 | 36.40 *^b^* | 360.00 |
|  |  |  | 6.60-12.50 | 3.03-9.44 | 5.30-17.10 | 16.30-55.50 | 173.00-506.00 |
|  |  | F | 11.75 | 5.55 | 9.60 | 27.40 | 318.00 |
|  |  |  | 8.90-13.90 | 2.20-9.87 | 3.60-17.00 | 8.50-53.20 | 171.00-503.00 |
|  | C3H/HeJ | M | 6.60 | 7.83 | 13.60 | 41.40 | 781.00 |
|  |  |  | 5.40-8.10 | 2.46-9.30 | 2.60-16.00 | 7.60-48.80 | 199.00-904.00 |
|  |  | F | 7.20 | 8.29 | 15.30 | 46.20 | 666.00 |
|  |  |  | 5.50-9.20 | 2.04-9.02 | 3.90-16.10 | 10.30-49.90 | 183.00-855.00 |
| Inter strain differences *^d^* | | | C3H/HeJ  *p*<0.05 | C3H/HeJ  *p*<0.05 | C3H/HeJ  *p*<0.05 | C3H/HeJ  *p*<0.05 |  |
| 9-11 months  (F=30) (M=30) | C57BL/6J | M | 8.70 *^c^* | 9.04 | 13.80 | 45.40 | 950.00 *^c^* |
|  |  |  | 2.70-14.00 | 8.27-9.91 | 12.20-15.00 | 40.30-48.50 | 418.00-1187.00 |
|  |  | F | 4.30 | 9.16 | 13.55 | 44.80 | 620.00 |
|  |  |  | 2.70-10.50 | 8.60-9.67 | 12.80-14.40 | 42.80-46.80 | 157.00-939.00 |
|  | 129SV/EV | M | 5.65 | 8.19 | 12.80 | 39.85 | 529.00 *^b^* |
|  |  |  | 2.70-8.10 | 3.90-10.04 | 6.40-15.30 | 18.40-47.70 | 127.00-683.00 |
|  |  | F | 5.55 | 9.32 | 13.90 | 43.85 *^b^* | 422.00 |
|  |  |  | 2.40-9.80 | 4.08-10.39 | 7.50-15.60 | 12.90-50.90 | 131.00-754.00 |
|  | C3H/HeJ | M | 6.20 | 8.37 | 13.70 | 41.60 | 779.00 *^b^* |
|  |  |  | 2.60-9.40 | 8.00-9.20 | 13.30-14.40 | 38.00-44.00 | 683.00-905.00 |
|  |  | F | 7.10 | 8.06 | 13.10 | 39.65 | 652.00 |
|  |  |  | 2.70-9.10 | 5.79-8.84 | 9.20-14.30 | 28.10-43.50 | 508.00-793.00 |
| Inter strain differences *^d^* | | |  |  |  |  | 129SV/EV  *p*<0.05 |

*^a^* WBC: White blood cells, RBC: Red blood cells, HGB: Hemoglobin, HCT: Hematocrit, PLT: Platelets;

*^b c^* Statistically significant intersex mouse strain different values: *p*<0.05 and *p*<0.001 respectively; *^d^* Statistically significant different values in the reported mouse strain vs the other strains.
